# Supplementary material for: Persistent frequent emergency department users with chronic conditions: A population-based cohort study
Source: PLoS One. 2020 Feb 12;15(2):e0229022. doi: 10.1371/journal.pone.0229022 (PMC7015381; doi:10.1371/journal.pone.0229022)
Supplement: S2 Table — (DOCX) [file pone.0229022.s002.docx]

|  | **ICD-9** | **ICD-10** |
| --- | --- | --- |
| **Dementia** | 290, 2941, 3310, 3312 | F00, F01, F02, F03, G30, F051, G311 |
| **Asthma** | 493 | J45 |
| **Chronic obstructive pulmonary disease** | 490-492, 494, 496 | J40-J44, J47 |
| **Congestive heart failure** | 428, 518.4 | I50, J81 |
| **Coronary heart disease** | 410-414 | I20-I25 |
| **Diabetes** | 250 | E10-E14 |
| **Epilepsy** | 345 | G40-G41 |
| **High blood pressure** | 401-405 | I10-I13, I15 |
