# Supplementary material for: Persistent frequent emergency department users with chronic conditions: A population-based cohort study
Source: PLoS One. 2020 Feb 12;15(2):e0229022. doi: 10.1371/journal.pone.0229022 (PMC7015381; doi:10.1371/journal.pone.0229022)
Supplement: Attachment — Submitted filename: Chiu_PLOSONE-Responses.docx [file pone.0229022.s004.docx]

**January 15^th^, 2020**

**PLOS ONE**

Revisions for “Persistent frequent emergency department users with chronic ambulatory care sensitive conditions: a population based cohort study”

Dear Dr Orueta,

We would like to thank you again, along with the reviewers for their second round of comments on our manuscript. We believe that the comments allowed us to improve the quality of our manuscript. Please find attached the revised version and the detailed responses to each of the comments.

We hope the revisions will be to your satisfaction and we look forward to hearing back from you.

Best regards,

Yohann M. Chiu, Ph.D.

Corresponding Author

Université de Sherbrooke

3001, 12e Avenue Nord

Sherbrooke, QC J1H 5N4

Phone: 819-346-1110, #70538

Email: [yohann.chiu@usherbrooke.ca](mailto:yohann.chiu@usherbrooke.ca)

# Editor’s comments

Most of the mentions of ACSC found in the title, text and tables are misleading. Almost universally, ACSC are referred to preventable hospitalizations or ED visits potentially avoidable. The population of this study are all the patients with any of a group of chronic diseases that visited ER. Such diseases could be considered as ACSC if they would have been the reason for such visits. Consequently, in order to avoid confusion for the readers, the term ACSC should be replaced by “chronic conditions” or other similar.

**Response**: We agree; since we did not investigate the reason for ED visit, we have updated the text with “chronic conditions” instead of “ambulatory care sensitive conditions”.

As reviewer 2 points out, I do not find a sufficient reason to exclude the patients with dementia. It seems plausible that such patients presented different health care needs to others subjects, but the same reason can be argued in relation to other diseases (for example HBP compared to asthma, COPD, or CHF) or age-groups.

**Response**: We have run our analyses including this time patients with dementia, although this does not change the results or the interpretations. We have updated the manuscript with those latest results.

The authors should also follow the recommendations of reviewer 1 about the statistical model, inclusion of measures of model fit, and presentation of the results.

**Response**: We have updated the methods and the results according to the comments of the reviewer 1 (see below for detailed answers).

# Besides, there are some minor errors in the draft. The description of the process to exclude patients presented in text (page 7) does not follow the same order as the one in figure 1; it seems to be a typo in the number and percentage of patients due to death. Also, in discussion (page 16, line 261) it is stated that “deprivation indices were not significant in our analyses”, in contradiction to the results of the study.

**Response**: The text for the description of the sample selection now follows Figure 1 (Lines 123-138); thank you for pointing out that. Regarding deprivation indices, it is true that they were significant in the occasional frequent use model, but they were not in the persistent frequent use model. We mentioned this difference in the discussion.

# Reviewer #1

I think the paper is improved with the changes. The purpose is clearer and the samples included in the analyses are also clearer. However, I continue to have some questions about the statistical approach and statistical conclusions, and the reporting of the results as well.

There is still an issue in these logistic regressions concerning the rarity of the outcome for the group ‘Persistent frequent users’, which comprise only 1/10 of 1% of the sample. As these models are inferential models intended to produce generalizable inferences about relationships between predictive factors and membership in a rare utilization group I think the models need more attention. Especially given the number of predictive variables (and their levels) considered in these models. (As an example, I wonder how many cases there are in the multidimensional cross-tabs of ‘age >-85 and ‘has ACSC diabetes’, given all the other predictors. One might assume that this multidimensional cell size is quite small.) I think that in order for the results from these models to be credible some additional sensitivity analyses are warranted. I recommend considering Firths’ penalized logistic regression (Puhr R, Heinze G, Nold M, Lusa L, Geroldinger A. Firth‘s logistic regression with rare events – accurate effect estimates and predictions? Statistics in Medicine 2017.).

**Response**: We thank the reviewer for this pertinent suggestion. It is true that, given the small prevalence of persistent frequent use, specialized models should be used. We have run the analyses using Firth’s penalized logistic regression and have modified the methods and results sections accordingly. However, the results do not change in regards to numeric values or interpretations (though the updated results in the manuscript are slightly different as they now include patients with dementia, as suggested by another reviewer).

Additionally I strongly recommend the variable and model screening processes be reported as well as some measures of model fit, at a minimum an R2 measure, and possibly also ROC/AUC or information criteria measures for model fit and precision in order to allow readers to gauge for themselves how these models fit, and potentially how incrementally important the identified predictors are relative to a baseline model.

**Response**: Area under the curve, R^2^, and BIC have been added to the analysis (Table 2). However, since the goal is to evaluate explicative factors for persistent frequent use and not to compare different models, we did not compare our models to a baseline model. Line 229

Reporting issues:

The statistical analysis section states that the first result is ‘prevalence of persistent frequent ED use’, however Table 1 does not contain that result (rather it reports ‘frequent users’). Is this a typo in the table?

**Response**: We agree that it was not clear; we reported the prevalence of persistent frequent ED use in Table 1 but did not mention it. We clarified it in the table. Lines 207-208

Pg 11, line 207-208 should specify if the results presented in Table 2 represent the results of 2 separate models comparing 1) occasional Frequent users to the entire sample and 2) comparing Persistent frequent users to the entire sample (vs comparing these two groups to each other) because it is still confusing. Additionally, the N’s should be reported in this table.

**Response**: Table 2 indeed presents results for two regression models: 1) occasional frequent users versus the entire sample and 2) persistent frequent users versus the entire sample. We have clarified this in the text and added the sample sizes in the table. Lines 215-217

What are the cells in Table 2 that contain a ‘-‘? Were those coefficients not estimable?

**Response**: Table 2 contains results for two models: 1) occasional frequent use and 2) persistent frequent use. Since both models went through an automatic variable selection process, the explicative variables included were not necessarily the same for both; in particular, there were more variables for the first model. The dash ‘-‘ means that a variable was not selected therefore not estimated for the persistent frequent use model. We clarified this point in a footnote of Table 2. Line 229

# Reviewer #2

The revised version of manuscript by Chiu YM et al. responded only partially to our concerns and also to the important concerns of other reviewers and to the Journal Requirements. In particular I continue to be convinced that not consider older people with chronic diseases such as dementia who, in my opinion, often could be considered as ambulatory care sensitive, may cause a bias in the full interpretation of the problem.

**Response**: Thank you for pointing out the possibility of a bias in the interpretations when excluding patients with dementia. We thus have run our analyses including them (n=12,189, 4.1 % of the total sample). Results and interpretations remain the same, though the material deprivation index is now significant in the logistic models. The sample selection, results and discussion are now updated. Lines 137; 155; 252
